# Investigation of 3D Printed Self-Sensing UHPC Composites Using Graphite and Hybrid Carbon Microfibers

**DOI:** 10.3390/s24237638

**Published:** 2024-11-29

**Authors:** Han Liu, Simon Laflamme, Bin Cai, Ping Lyu, Sri Sritharan, Kejin Wang

**Affiliations:** 1 Department of Civil, Construction, and Environmental Engineering, Iowa State University, Ames, IA 50011, USA; liuhan@iastate.edu (H.L.); binc@iastate.edu (B.C.); pinglyu@iastate.edu (P.L.); sri@iastate.edu (S.S.); kejinw@iastate.edu (K.W.); 2 Department of Electrical and Computer Engineering, Iowa State University, Ames, IA 50011, USA

**Keywords:** 3D printing, additive manufacturing, UHPC, cementitious, structural health monitoring, self-sensing, strain sensing, carbon microfiber

## Abstract

This paper explores the development of 3D-printed self-sensing Ultra-High Performance Concrete (UHPC) by incorporating graphite (G) powder, milled carbon microfiber (MCMF), and chopped carbon microfiber (CCMF) as additives into the UHPC matrix to enhance piezoresistive properties while maintaining workability for 3D printing. Percolation curves were established to identify optimal filler inclusion levels, and a series of compressive tests, including quasi-static cyclic, dynamic cyclic, and monotonic compressive loading, were conducted to evaluate the piezoresistive and mechanical performance of 29 different mix designs. It was found that incorporating G powder improved the conductivity of the UHPC but decreased compressive strength for both mold-cast and 3D-printed specimens. However, incorporating either MCMF or CCMF into the UHPC resulted in the maximum 9.8% and 19.2% increase in compressive strength and Young’s modulus, respectively, compared to the plain UHPC. The hybrid combination of MCMF and CCMF showed particularly effective in enhancing sensing performance, achieving strain linearity over 600 με. The best-preforming specimens (3G250M250CCMF) were fabricated using 3 wt% of G, 0.25 wt% of MCMF, and 0.25 wt% of CCMF, yielding a maximum strain gauge factor of 540, a resolution of 68 με, and an accuracy of 4.5 με under axial compression. The 3D-printed version of the best-performing specimens exhibited slightly diminished piezoresistive and mechanical behaviors compared to their mold-cast counterparts, yielding a maximum strain gauge factor of 410, a resolution of 99 με, and an accuracy of 8.6 με.

## 1. Introduction

It is possible to functionalize cementitious materials with strain-sensing properties conferred through the incorporation of conductive or semiconductive fillers [1]. Such self-sensing cementitious materials have gained significant interest in the research community due to their potential applications in the structural health monitoring (SHM) of civil infrastructure [2]. This is particularly because the self-sensing units offer mechanical robustness comparable to that of the infrastructure, and they may also be utilized in hostile environments. The sensing principle is generally based on leveraging the material’s piezoresistivity [3,4] to provoke measurable changes in the electrical signal upon deformations [5]. This sensing mechanism allows for the detection of strain, stress, cracks, damage, temperature, and humidity by appropriately mapping to variations in electrical resistivity, which is useful in enabling the continuous monitoring of structural integrity and reliability while maintaining or even enhancing the component’s mechanical properties and durability [6,7].

Concrete has long been the most commonly used construction material due to its cost efficiency, good durability, and adaptability [8,9]. Yet, the need for materials that offer enhanced performance and functionality has become increasingly critical as resiliency and sustainability demands on infrastructure have evolved [10,11]. Of interest to this research is Ultra-High Performance Concrete (UHPC), first introduced in 1994 [12], that raised significant interest in the last two decades due to its superior mechanical properties, durability, and versatility in complex construction applications [13,14]. It has been used in the construction of bridges [15], buildings [16], and structural repairs [17,18], with known advantages in high-performance load-bearing applications [19] and for producing longer structural lifespans [20,21].

UHPC possesses a dense microstructure that enhances mechanical strength and improves durability [22]. This microstructure offers opportunities to control the filler to provide strain-sensing capabilities without significantly compromising the mechanical performance. In addition, its low water-to-binder ratio, combined with the use of fine powders and fibers, allows for better control over the distribution and orientation of conductive fillers. The fabrication of self-sensing UHPC has been widely studied, for example, through the incorporation of carbon black [23], carbon nanotubes [24], carbon microfiber [25], graphite [26], metal oxides [27], graphene nanoplatelets [28], steel fibers [29,30], etc.

Because of these properties, UHPC has also been proposed for 3D printing (3DP) applications as a solution to the weak tensile strength of typical 3D printed cementitious structures [31,32]. 3DP structural materials from UHPC offer substantial benefits, including higher material efficiency, lower costs, faster construction speed, the elimination of formworks, reduction in labor, reduced environmental impact, and enhanced worker safety [33,34,35]. However, using UHPC for that purpose can be challenging because of the material rheology that reduces printability, and the uniformity in the distribution of its filler correlates with the stability and repeatability of its pressure-sensitive properties [3,36]. Recent advances have explored various fiber and textile reinforcements to enhance the mechanical performance of UHPC. For instance, the inclusion of steel fibers has been shown to improve tensile strength and toughness, yet negatively affecting flowability and extrudability [37]. Synthetic fibers, such as polypropylene and polyethylene, have been investigated for their ability to enhance crack resistance without significantly impacting printability [38]. Textile reinforcements, such as alkali-resistant glass, have shown promise in enhancing deformation capacity, deflection control, and the overall structural integrity of printed UHPC components [39].

To fully empower UHPC as a material for 3DP, one needs to be able to guarantee adequate structural performance for each print. This can be achieved by conferring strain-sensing capability to the material. Such self-sensing UHPC could be also useful for other SHM applications, for example, to monitor load-bearing conditions and structural integrity under operational loads [4,40]. In prior work, we have studied 3DP self-sensing cementitious materials using a cement paste [41]. The results demonstrated satisfactory strain-sensing properties, but limited mechanical performance. In this paper, we explore the use of UHPC without steel fiber inclusion to print cementitious sensors and improve the mechanical properties, while maintaining the capability to monitor strain.

Graphite (G) powder, milled carbon microfiber (MCMF), and chopped carbon microfiber (CCMF) were incorporated into the UHPC matrix to enhance the piezoresistive properties necessary for effective self-sensing performance. These materials were selected based on previous studies, including our study on 3DP cementitious self-sensing paste [25,41], that addressed the limitations of using single CMF fillers. Specifically, G powder has been extensively studied for its ability to impart electrical conductivity to cement-based composites [42], while MCMF functions as a short-range conductor and CCMF as a long-range conductor. The hybrid combination of MCMF and CCMF has shown significant effectiveness in establishing a stable conductive network within cement and concrete materials [25,43], transforming them into functional materials capable of monitoring the behavior of structural members in real-time.

Thus, the novelty of this paper resides in the study of 3D printable self-sensing UHPC as a mechanically stronger alternative to 3D printed self-sensing cement paste. A total of 29 different mix designs are investigated to produce this functionalized material. Electrical percolation curves were established to characterize the effects of varying MCMF and CCMF inclusion levels, as well as their hybrid synergistic effects on the electrical conductivity and piezoresistivity of the UHPC. A series of compression tests consisting of quasi-static cyclic loading, dynamic cyclic loading, and monotonic compressive loading was performed to characterize and evaluate both the sensing properties in terms of linearity, strain sensitivity, stress sensitivity, resolution and accuracy, as well as the mechanical properties in terms of compressive strength and Young’s modulus. 3DP specimens were fabricated using identified optimal mix formulations and compared to their mold-cast counterparts to assess the performance differences attributable to the manufacturing processes.

The rest of the paper is organized as follows: Section 2 provides the background on the composite of UHPC, the material properties of the conductive filler, the electrical percolation mechanism, the fabrication process, and the derivation of the electromechanical model. Section 3 describes the quality inspection and experimental methodology. Section 4 presents and discusses results from the experimental investigation and evaluates the mechanical and sensing performance. Section 5 concludes the paper.

## 2. Background

### 2.1. UHPC

UHPC is a composite material typically formulated with cement, fine aggregates, silica fume, high-range water-reducing admixture (HRWR), metal fibers, water, and additional chemical admixtures [14]. Different combinations of these materials can be used depending on the application and supplier. The UHPC employed in this study is the commercially available Ductal^®^, with its typical composition listed in Table 1 [44]. An important challenge in 3DP UHPC is its highly dense matrix that contains fine particles such as silica fume and fine aggregates, which impacts its flowability and workability. As a result, the inclusion of steel fibers was left to future work to ensure a smooth and continuous 3DP process, because their presence could provoke blockages and irregular depositions [37]. Without steel fiber, the mixtures used in this study exhibit lower compressive strength and Young’s modulus compared to conventional UHPC. The inclusion of steel fibers would require the modification of the UHPC formulation, optimization of extrusion parameters, and/or re-design of the extruder in order to enhance fiber distribution without compromising print quality.

### 2.2. Carbon Microfibers (CMF)

Self-sensing cementitious materials are generally composed of a cementitious matrix and conductive fillers. The cementitious matrix serves as a binder and provides the mechanical properties of the composite. The conductive fillers, in contrast, consist of materials that impart the electrical conductivity necessary to reach electrical percolation and may also enhance the mechanical performance. The conductive fillers considered in this study include G powder (TITGGI 400 mesh), as well as the MCMF (C M150-4.0/240-UN) and CCMF (C C6-4.0/240-G100) both purchased from SGL Carbon (201 Technology Drive, AR 71932 Arkadelphia, USA) [45], selected based on findings in [25,41]. The G powder and both types of CMF exhibited good feedability, dispersibility, and compatibility with the cementitious material and were, therefore, used as received, without further modification or purification. Their material properties are listed in Table 2.

**Figure 1:**
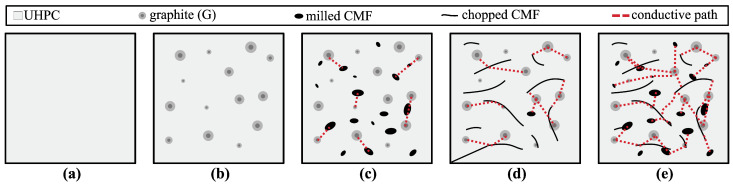
Illustration of the electrical percolation process: (**a**) UHPC-only (without fiber); (**b**) G-only; (**c**) G + MCMF; (**d**) G + CCMF; (**e**) G + MCMF + CCMF.

The electrical percolation mechanism of self-sensing cementitious material is illustrated in Figure 1. Typical cement-based composites are porous, and their electrical conductivity mainly depends on the interconnected pores and ionic conduction [46]. However, UHPC has relatively very low porosity and no interconnected pores, thus resulting in poorer electrical conduction (Figure 1a). G is a form of carbon that has a layered and planar structure in which carbon atoms are arranged in a hexagonal lattice. This structure facilitates the presence of free electrons, imparting inherent electrical conductivity to the UHPC composite due to its high electron mobility (Figure 1b) [47]. G flakes tend to form conductive networks when their concentration exceeds a certain threshold. The high packing density and reduced porosity of UHPC facilitate closer contact between G particles, allowing electrons to both tunnel across small gaps and hop from one conductive particle to another within the UHPC matrix [42]. The enhanced electron interaction lowers the percolation threshold of the composite, meaning that electrical percolation can be achieved with lower amounts of filler while maintaining the flowability and workability of the fresh composite. Additionally, the formation of dense and interconnected calcium silicate hydrate (C-S-H) phases during the hydration process of UHPC can embed the G particles, mitigating the possible clumping of other conductive fillers and promoting a more homogeneous functional filler distribution [48,49].

CMF particles contribute significantly to the creation of conductive paths. MCMF, characterized by their shorter and finer structure and high surface area, interweave with the G flakes, effectively filling the gaps and interstitial spaces, thus enhancing the overall electrical conductivity by creating additional conductive paths (Figure 1c). CCMF, characterized by a higher aspect ratio (defined as the ratio of the longer to the shorter dimension of the particles) and elongated shapes, bridge larger gaps within the UHPC matrix, forming an interconnected network of conductive paths (Figure 1d).

Yet, the high aspect ratio of conductive fibers is also associated with a higher propensity for entanglement and agglomeration, which adversely impacts the signal repeatability of self-sensing cementitious materials [50]. In contrast, smaller aspect ratio particles and powders reduce the risks of debonding and reorientation [51]. As a result, dual-doping strategies that combine particles of different aspect ratios have been widely reported in the literature as a means to create a more robust and efficient conductive network, improving both the sensitivity and repeatability, while also enhancing the mechanical properties of functionalized materials [23,24,52,53]. Specifically, dual-doping with both MCMF and CCMF has a synergistic effect, wherein the microscopic spaces between adjacent conductive fibers are occupied by MCMF, while CCMF bridges larger gaps, resulting in a multiphasic and multiscale conductive network with enhanced connectivity (Figure 1e).

### 2.3. Composite Mixtures

**Figure 2:**
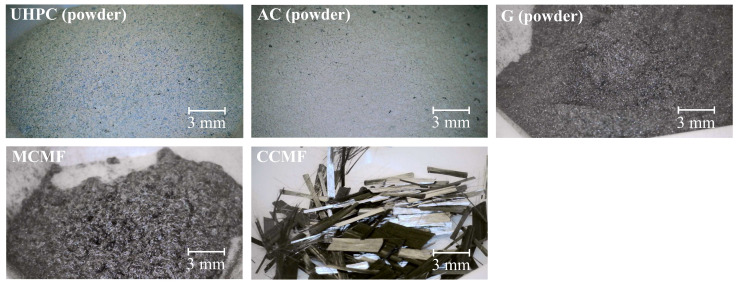
Magnified pictures of dry mixture components.

The self-sensing UHPC composite mixture used in this study includes commercially available Ductal^®^ UHPC premix powder, alumina cement (AC—also known as calcium aluminate cement) powder that contains a high concentration of aluminate phases, specifically calcium aluminate (CaAl_2_O_4_), monocalcium aluminate (CaAl_2_O_4_), dicalcium aluminate (CaAl_4_O_7_), along with mayenite (Ca_12_Al_14_O_33_) and ferrite phases (CaAlFeO_4_), G, MCMF, CCMF, water, and HRWR (MasterGlenium 7920). Figure 2 presents magnified images of these solid components, while their material properties, including density, conductivity, and aspect ratio, are detailed in Table 3.

The mix designs for UHPC-only, G-filled-only, single MCMF-filled, single CCMF-filled, and hybrid MCMF + CCMF-filled UHPC specimens are listed in Table 4. The mixture designs identification is constructed as follows: except for mixtures used for 3DP that start with ‘3DP-’, the first term indicates the weight percentage of G, and the subsequent terms indicate the weight percentages (×10^3^) of MCMF and/or CCMF, using only ‘M’ for brevity of the naming for hybrid formulations. All specimens are fabricated using 1380 g of UHPC, 120 g of AC powder, and 1.0 wt% of HRWR-to-binder (HRWR/b) ratio. The use of the Ductal^®^UHPC premix powder, AC powder, and G powder affects the rheological properties of the UHPC mixture, impacting its pumpability and extrusion characteristics. The mixture formulations are based on previously designed UHPC mixes and are optimized to meet the requirements for feedability, extrudability, flowability, and buildability in 3D printing.

Here, the inclusion of AC powder promotes early strength development due to its rapid setting characteristics, which reduces slump and mitigates shape distortions (e.g., tilt, twist, or collapse) as subsequent layers are deposited. It also helps to minimize shrinkage, which can lead to cracking and dimensional inaccuracies in the final print [54]. The use of polycarboxylate-based HRWR improves workability and flowability, reduces particle agglomeration, and facilitates the dispersion of conductive fillers throughout the mixture, resulting in smooth extrusion through the printer nozzle and ensuring strong layer adhesion [23,55]. The water-to-binder (b/w) ratio is maintained at a low yet sufficient level to ensure proper hydration of UHPC while preserving flowability and printability. This minimizes the fluctuations in electrical conductivity caused by the electrolytic pore solution and the re-agglomeration of conductive fillers during mixing [56]. Additionally, maintaining a low b/w ratio helps minimize changes in shrinkage-recovery behavior under cyclic loading, issues that are exacerbated by higher water-to-binder ratios [57].

G powder is introduced at an initial concentration of 15 g (1 wt%) and incrementally increased in 15 g steps up to 75 g (5 wt%), with additional increments to 120 g (8 wt%) and 240 g (16 wt%). These increments are designed to investigate the critical concentration of G necessary to achieve electrical percolation, which occurs when a continuous conductive network is established throughout the matrix. The inclusion of MCMF starts at 0.468 g (0.03125 wt%) and doubles until reaching 15 g (1 wt%), following a similar procedure for CCMF. All dosages presented in this study are given as a weight percentage of the binder. The hybrid specimens’ inclusion levels were selected based on the percolation curves obtained from the MCMF and CCMF specimens, as discussed later in Section 4. A UHPC-only specimen (0G0CMF) was also prepared as a benchmark for comparison. Note that the w/b ratio increases with the increased content of G powder due to the particles’ high surface area that absorbs water and requires additional water to maintain workability, homogeneous dispersion, and proper hydration of the mixture. The w/b ratio for cases of single MCMF filled, single CCMF filled, and hybrid MCMF+CCMF filled UHPC specimens was maintained constant within their respective categories to ensure consistency in the evaluation of their properties.

### 2.4. Fabrication Process

**Figure 3:**
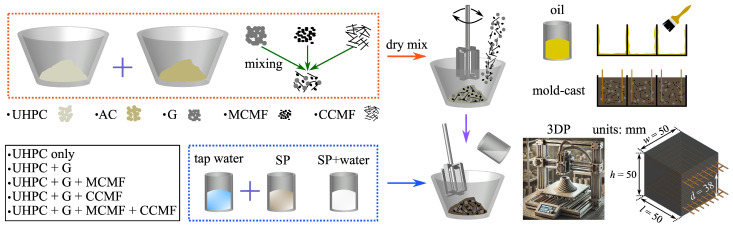
Fabrication process of the mold-cast and 3D-printed self-sensing UHPC specimen.

The fabrication and curing of UHPC specimens mostly followed the ASTM C1856/C1856M-17 guidelines [58], but with minor adjustments to accommodate the specific characteristics of the UHPC mix and 3D printing process. The fabrication process of the specimens is illustrated in Figure 3. First, all solid components (i.e., UHPC, AC, G, and CMF) were weighed according to the designed mixture proportions (Table 4) and dry-mixed using a Hobart mixer (H-3841, 5-Qt, Hobart Corporation, Troy, OH, USA, ASTM D5581 compliant) for two minutes to break up particle agglomerations and ensure uniform dispersion of the particles and fillers throughout the mix. Next, the HRWR/water solution was prepared by adding 1 wt% HRWR, relative to the binder weight, to the water. This solution was then added to the dry mixture and mixed for 12 to 15 min at a low rotary speed of 136 RPM, followed by 3 min at a high speed of 256 RPM, resulting in a total mixing time of 15 to 18 min. The mixing time varied within this range depending on the desired consistency and workability, which could differ based on the specific mixing proportions to achieve a fresh, homogeneous mixture with uniform filler dispersion.

Finally, mixtures were cast into pre-oiled cubic molds (50.8 mm × 50.8 mm × 50.8 mm) using a double pour method, with tamping after each pour to release air bubbles and ensure uniform compaction. Two copper wire meshes (wire diameter of 1.3 mm, mesh openings of 5.33 × 5.33 mm^2^, overall width of approximately 45 mm, and overall length of approximately 60 mm) were embedded in parallel with a spacing interval *d* of approximately 38 mm, as shown in Figure 3, during the casting of the specimens to serve as electrodes for measuring changes in electrical resistivity during curing and subsequent mechanical testing. A trowel was used to level the surface of the mold-cast UHPC specimen for a smooth finish. Sets of four specimens were fabricated under the 29 different mix designs (Table 4), with varying inclusion levels of G and CMF, for a total of 116 cast specimens. The quality of the dispersion of the G and CMF in the UHPC matrix was examined through resistance measurements, signal stability and drift, and frequency response analysis, as discussed in greater detail in Section 3.1.

**Figure 4:**
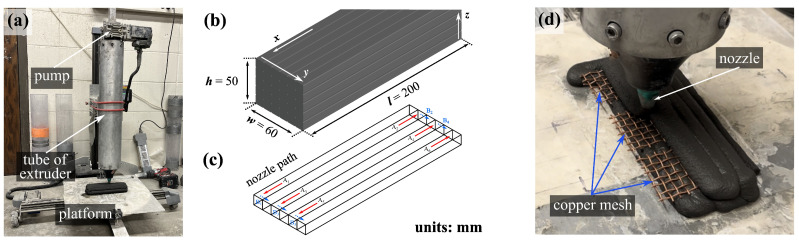
(**a**) Picture of the 3D printer; (**b**) schematic illustration of the 3D-printed rectangular prism showing the overall geometry dimension and the directional description of 3DP process; (**c**) directional description of the nozzle path; (**d**) picture of the 3DP process.

The top three strain-sensing performing mix designs identified from testing of the mold-cast specimens, as discussed in greater length later, were selected for the 3DP investigations, namely 3DP-3G125M500CCMF, 3DP-3G250M250CCMF, and 3DP-3G250M500CCMF. Ice water was utilized during the mixing process to cool the mixture, thereby extending the workable time and maintaining the fluidity and workability necessary for the printing process. The printed geometry consisted of a rectangular prism composed of five layers, each formed by six adjacent filaments. Each filament had a constant length of 200 mm, width of 10 mm governed by the material flow and spreading at deposition, and thickness of 10 mm, resulting in an overall outer dimension of approximately l×w×h=200×60×50 mm^3^ (Figure 4b), allowing for three 50.8 mm cubic specimens to be cut off from it, totaling nine 3D-printed specimens for the selected three different mix designs.

The 3DP model was sliced using the 3DP software Simplify3^®^ version 4.1, and the printing direction is shown in Figure 4b, where the *x* axis is defined as the filament direction in which the nozzle moves such that all filaments are parallel to the *x* axis, the *y* axis is perpendicular to the filament direction (to *x*) along the layer plane, and the *z* axis is along the vertical direction perpendicular to the layers. The moving path of the nozzle, detailed in Figure 4c, initiates with the nozzle moving along a predetermined line parallel to the *x* axis (A_1_) to complete the deposition of the first filament. The nozzle then progresses by a distance equivalent to the filament width in the *y* direction (B_1_), continuously extruding to initiate the second filament along the A_2_ path. This process is repeated until the completion of A_6_ filament extrusion, with the layering process continuing until the entire prism is fully printed.

An extrusion-based commercial 3D clay printer (3D Potter 7) was used for 3DP. It has a resolution in the *x* and *y* axis of 0.15 mm, a resolution in the *z* axis of 0.7 mm, a printing envelope x×y×z = 415 × 405 × 500 mm^3^, and a maximum printing speed of 200 mm/s. The overall setup of the 3D printer with key annotations is shown in Figure 4a. The printing process involved the following: (1) Feeding—the fresh UHPC composite was manually placed into the extruder tube in three consecutive pours, with each layer being rodded approximately 10 times to eliminate air pockets, and the filled tube was then installed on the printer; (2) Configuring—a circular nozzle with a diameter of 8 mm was attached to the extruder, the extrusion rate was set to 2100 mm^3^/s, the printing speed (the movement speed of the printer head and platform) was set to 20 mm/s, and a stand-off distance of 10 mm. The vertical shift was set to 10 mm for each subsequent layer, thus producing a 10 mm layer height. This adjustment was based on prior optimization experiments to maintain the desired overall print dimensions, prevent layer overlap or excessive compression, and ensure proper layer adhesion and surface finish quality [59]; (3) Pre-printing—the 3D printer was operated to print a preliminary filament perimeter, which was discarded to purge any air trapped in the nozzle and extruder, thereby ensuring consistent and smooth extrusion; (4) Printing—the UHPC composite was systematically deposited in sequential strips and layers according to the predefined path (Figure 4b) to complete the designed geometry. Three sets of copper meshes were inserted after the first and fourth layer of printing to form parallel electrodes, thus spaced by approximately d=30 mm, as shown in Figure 4d. This arrangement was dictated by the layer-based deposition process, which constrained the electrode placement to align with printed layers. A slightly different spacing interval d=38 for the electrodes was used for the mold-cast specimens, and taken into account in computing resistivity using Equation (1).

Upon the completion of casting or printing, specimens were covered with plastic sheeting to prevent the rapid evaporation of water and thus minimize shrinkage. Specimens were initially cured in a laboratory environment at a constant temperature of 25 °C for 48 h. Afterward, the mold-cast specimens were demolded, and both mold-cast and 3D printed specimens were moved into a standard moisture curing room (23 ± 2 °C and 95 ± 3% RH) for an additional 26 days, totaling a curing period of 28 days. Before conducting electrical and piezoresistive measurements, all specimens were dried at 60 °C in an oven for 24 h to eliminate excess moisture that could otherwise skew electrical resistivity measurements.

### 2.5. Electromechanical Model

The strain-sensing capability of the functionalized UHPC stems from the material’s piezoresistive effect. Self-sensing cementitious materials fabricated with conductive dopants can be modeled as a resistor of nominal electric resistance $R_{0}$:

(1) $$R_{0}=\rho \frac{A}{d}$$

where ρ is the resistivity, *A* = w·l = 2500 mm^2^ is the cross-section area, and *d* = 38 mm is the distance between the integrated copper electrode nets as annotated in Figure 3. Assuming small deformation and uniaxial stress under compression along the *z* direction (Figure 4b), the fractional change in resistance (FCR) is taken as follows:

(2) $$\mathrm{FCR}=\frac{\Delta R}{R_{0}}=\frac{R-R_{0}}{R_{0}}$$

where ΔR is the variation in resistance, and $R_{0}$ is the initial resistance before compression. By differentiating the natural log of Equation (1), the FCR can be written as a function of the compression strain ε:

(3) $$\mathrm{FCR}=\frac{\Delta \rho }{\rho }+\frac{\Delta e}{e}-\frac{\Delta A}{A}=\frac{\Delta \rho }{\rho }+\left(1+2\nu \right)\varepsilon =\lambda \varepsilon$$

where ν is the Poisson’s ratio of the material, and ε is the strain between the electrodes taken negative in compression. The strain gauge factor $\lambda_{\mathrm{strain}}$ can be written as follows:

(4) $$\lambda =\frac{\mathrm{FCR}}{\varepsilon }=\left(1+2\nu \right)+\frac{\Delta \rho }{\rho }\frac{1}{\varepsilon }$$

where $\frac{\Delta \rho }{\rho }$ is the piezoresistive effect. The piezoresistive effect is approximately null for typical foil gauges but can be quite significant around electrical percolation for functionalized cementitious materials.

## 3. Experiments

### 3.1. Measurement

Percolation curves were constructed from resistance measurements taken using a two-probe alternative current setup at 28 days. This alternative current setup was selected because of the expected high resistivity and low conductivity of the specimens [60]. Specimens were subjected to a similar curing process but were temporally removed from the curing room for measurements and then returned to continue curing.

Resistance measurements were conducted using an LCR meter (Agilent 4263B, Agilent Technologies, Santa Clara, CA, USA) configured using the resistance-reactance (R-X) model with a measuring frequency of 1 kHz driven in LabVIEW to characterize the intrinsic polarization drift caused by either the dielectric nature of cementitious composites [61,62] or the direct piezoelectric effect [63,64]. The selection of the measurement frequency of 1 kHz was specifically aimed at minimizing the dielectric effect of the cementitious matrix and mitigating the issues related to electrode polarization and reactance [60,65]. This is because the use of embedded electrode meshes significantly reduces contact resistance at the interface between the electrode and the cementitious matrix by improving electrical contact and distributing the electrical load more evenly [66].

To examine the filler dispersion quality, frequency response analysis was conducted by measuring impedance across a range of frequencies, from 100 Hz to 100 kHz, to assess the frequency-dependent behavior of the conductive filler network. This frequency sweep characterizes changes in filler network connectivity and identifies any inconsistencies in dispersion by analyzing how impedance and phase angle vary with frequency. Consistent impedance across frequencies suggests well-dispersed conductive pathways, while fluctuations indicate potential agglomeration or uneven filler distribution within the matrix.

Any specimen exhibiting erratic fluctuations, noise values outside an acceptable range, inconsistent impedance across frequencies, or significant deviations in resistance from the average of the three specimens within its category was discarded as a sign of poor quality in particle dispersion and/or electrode embedment and was re-fabricated.

Signal quality was assessed using the signal-to-noise ratio (${\mathrm{SNR}}_{dB}$) and mean absolute error (MAE):

(5) $${\mathrm{SNR}}_{dB}=10\cdot \log_{10}\left(\frac{P_{\mathrm{signal}}}{P_{\mathrm{noise}}}\right)$$

(6) $$\mathrm{MAE}=\frac{\sum_{i=1}^{z}\left|x_{{\mathrm{true}}_{i}}-x_{{\mathrm{est}}_{i}}\right|}{z}$$

where ${\mathrm{SNR}}_{dB}$ is in decibels, $P_{signal}$ and $P_{noise}$ in Equation (5) are the power of signal and noise, respectively, $x_{true_{i}}$ and $x_{est_{i}}$ are the true and measured values, respectively, and *z* is the total number of samples collected.

### 3.2. Compression Tests

**Figure 5:**
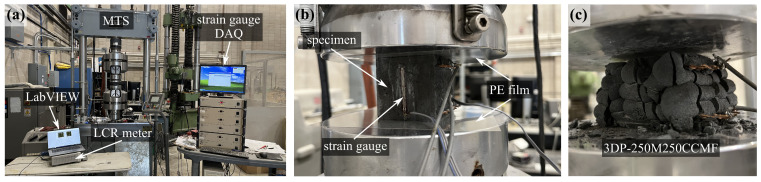
Pictures showing (**a**) overall experimental setup used to characterize sensing properties; (**b**) closeup view on a tested specimen; (**c**) 3DP specimen after brittle failure.

The strain sensing performance of the self-sensing UHPC specimens with different mix designs is characterized and evaluated through a series of load-control axial compression tests. Figure 5a shows the overall experimental test configuration. The loads were applied using a closed-loop servo-hydraulic testing machine (MTS model 312.41 with a TestStar IIm controller, MTS Systems Corporation, Eden Prairie, MN, USA ), equipped with 647 hydraulic wedge grips clamping a compression platens (model 643.15B-03) with a bearing unit stress of 689 MPa (100,100 psi). Figure 5b is a close-up view of the front side of the UHPC specimen, instrumented with strain gauges connected to a data acquisition system (DAQ). Specifically, the front and back surfaces were ground to remove irregularities and cleaned with isopropyl alcohol to create a bonding area. Two resistive strain gauges (TML FLA-30-11-1LJCT, A516912, RG24J, nominal resistance 120 ± 0.5 Ω, gauge length 30 mm) were installed on the prepared surfaces using non-conductive strain gauge adhesive (CN Cyanoacrylate) and then coated with polyurethane (Micro M-COAT A) for mechanical and electrical protection. These gauges were used to measure the actual compressive strain experienced by the specimen, which was then used to calibrate the strain data extracted from the MTS. Additionally, two polyethylene (PE) films were placed on both the top and bottom sides of the specimen, between the specimen and the loading platen, to provide electrical insulation. The wires were also secured with electrical tape to ensure proper electrical insulation.

Three different loading protocols, comprising quasi-static cyclic loading, dynamic cyclic loading, and monotonic compressive loading until failure, were designed and performed, each with a preload of 100 N applied to the specimen prior to testing to eliminate initial deformation. The quasi-static cyclic loading test involved applying axial compression loads along the longitudinal direction of the specimen through three sets of five-cycle harmonic excitation at 0.1 Hz. The load magnitudes for each set were 4.44 kN (1 kip), 8.88 kN (2 kips), and 17.79 kN (4 kips), corresponding to 1.72 MPa (0.25 ksi), 3.44 MPa (0.5 ksi), and 6.90 MPa (1 ksi), respectively. These load magnitudes were deliberately selected to remain below typical UHPC testing levels, ensuring that the test conditions remained within the linear elastic range of the material. This is particularly important for the self-sensing UHPC specimens fabricated in this study, which exclude steel fibers and are expected to have lower compressive strength and Young’s modulus. Testing at these reduced magnitudes allowed for a more accurate evaluation of the material’s sensing capabilities and response under controlled low-stress conditions.

The dynamic cyclic loading tests were performed by applying axial compression loads through five sets of five-cycle harmonic excitations at a loading magnitude of 8.88 kN (2 kips), with the loading frequency range from 0.2 Hz to 1 Hz in 0.2 Hz increments for each set, within the typical dynamic frequency response range of larger scale civil engineering structures [67,68]. A 0.5-second pause was applied after each dynamic set to accommodate machine adjustments. As for the monotonic compressive loading test, the uniaxial compression load was applied at a constant loading rate of 1.8 kN/s (0.4 kips/s) yielding a constant stress rate of 0.689 MPa/s (0.1 ksi/s) as per ASTM C109/C109M-20 [69]. The test was stopped when the maximum displacement reached 2.54 mm (0.1 in), at which point visible specimen failure or a significant reduction in load-carrying capacity, indicative of specimen failure, was observed. Figure 5c shows the picture of a 3D-printed UHPC specimen after brittle failure. The UHPC specimen exhibited a brittle failure mode with a rapid loss of load-carrying capacity, with visible cracks propagating through the surface and crushing occurring in the central region, behaviors typical of UHPC under compressive stress.

Load and displacement values were recorded at 20 Sample/second (S/s), and resistance values were simultaneously recorded at 10 S/s using the LCR meter (Agilent Technologies, Santa Clara, CA, USA). Data from the strain gauges were recorded using a System 5000 Scanner (Model 5100B, Vishay Precision Group, Malvern, PA, USA, resolution 1 με) configured to sample data at a rate of 1000 S/s. All tests were conducted in the laboratory under constant temperature conditions, and at least three specimens were tested for each mix design, with additional specimens tested as needed to ensure the consistency and reliability of the data.

## 4. Results and Discussion

### 4.1. Percolation Curves

**Figure 6:**
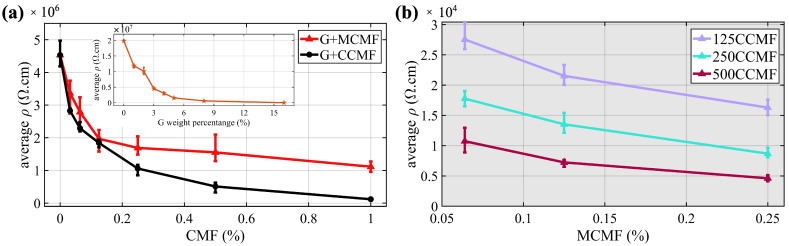
Percolation curves obtained from 28-day resistivity measurements for (**a**) single MCMF versus CCMF doping with the inset showing the percolation curve for G filling; (**b**) dual doping (MCMF + CCMF).

Figure 6a presents the 28-day three-specimen averaged resistivity ρ computed using Equation (1) as a function of inclusion levels, with the error bars representing the full range of resistivity values across the three measured specimens. The percolation study started with an investigation of the effect of G inclusions (inset of Figure 6a). The results showed that the resistivity of the UHPC specimens significantly decreased with increasing G content, particularly at lower percentages from 0 wt% to 5 wt%, with the most notable reduction occurring within the initial 3 wt% of G inclusions. Following these results, a 3 wt% dosage was selected to fabricate CMF-doped specimens.

The percolation curves for MCMF and CCMF are compared in Figure 6a, and exhibit a significant decrease in resistivity at low concentrations, with the most notable reduction in resistivity occurring within the range of 0.032 wt% to 0.25 wt% for MCMF and 0.0125 wt% to 0.5 wt% for CCMF, causing decreases in resistivity of 71% and 205%, respectively. The percolation behavior of MCMF (red line) differs from that of CCMF (black line) by exhibiting a more gradual decrease in resistivity, attributable to the distinct aspect ratios of the fibers.

From these results, the doping levels of 0.064 wt%, 0.125 wt%, and 0.25 wt% for MCMF, and 0.125 wt%, 0.25 wt%, and 0.5 wt% for CCMF were selected to span the electrical percolation thresholds. Percolation curves for hybrid dopants are plotted in Figure 6b. Results show that dual doping (MCMF + CCMF) provides higher conductivity and further accelerates percolation compare to single doping.

### 4.2. Quasi-Static Cyclic Load Tests

**Figure 7:**
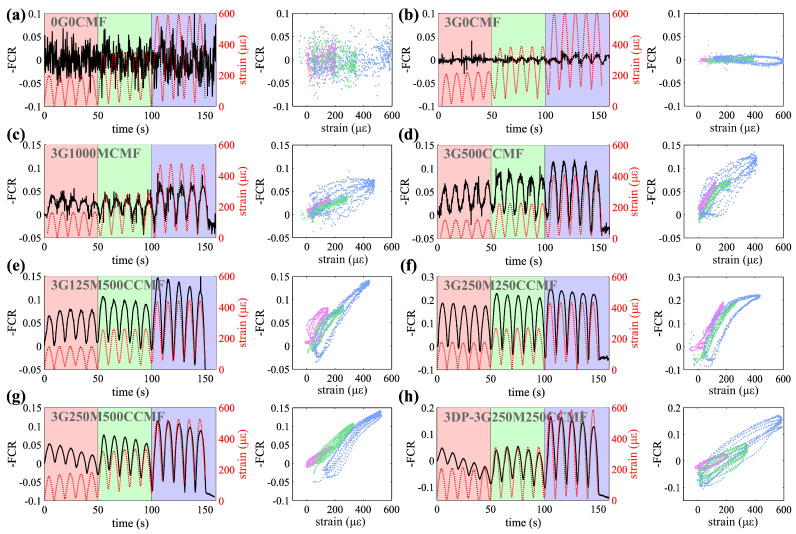
Strain sensing performance for key mix designs under quasi-static cyclic loading test for specimens (**a**) 0G0CMF; (**b**) 3G0CMF; (**c**) 3G1000MCMF; (**d**) 3D500CCMF; (**e**) 3G125M500CCMF; (**f**) 3G250M250CCMF; (**g**) 3G250M500CCMF; (**h**) 3DP-3G250M250CCMF. I don’t think we used hyphen in this figure. All symbols are minus sign. Same as below.

Figure 7a–h presents the electrical signal compared against the strain gauge-calibrated MTS strain measurements for representative specimens of key mix designs that exhibited the best sensing performance within their respective dopant categories, where the data measured under the compression loads of 4.44 kN, 8.88 kN, and 17.79 kN are distinctly represented by the pink, green, and purple colors, respectively. The presented strain values were extracted from the MTS machine and calibrated using strain gauge data, while the stress was calculated based on the applied compression load and the cross-sectional area. Resistance data are presented after being detrended to eliminate intrinsic polarization drift. The results from the quasi-static cyclic load tests were used to compute the SNR and MSE values for each specimen listed in Table 5, which summarizes the sensing performance.

It can be observed from Figure 7 that the UHPC-only specimen (0G0CMF) failed to provide meaningful measurements. The addition of G significantly reduced noise in measurements. Despite this improvement, the G-filled specimen still lacks strain-sensing sensitivity, as indicated by the low FCR magnitude under the compression, with the FCR values primarily attributed to ionic conduction [2]. Specimens with either MCMF or CCMF inclusions exhibited a substantial improvement in strain sensing capabilities, as indicated by the reduction in noise levels, reflected in the tighter clustering of data points in the FCR versus strain curves. The increase in the FCR magnitude suggests an improvement in the strain-sensing sensitivity. In comparison to the single MCMF or CCMF-filled specimens, the hybrid MCMF + CCMF-filled specimens exhibited further improved signal coherence and higher strain sensitivity. This improvement is particularly evident in specimens 3G250M250CCMF and 3G250M500CCMF. The 3D-printed dual-doped specimen maintained a sensitivity and stability comparable to that of the cast counterparts.

A cross-comparison of performance metrics in Table 5 reveals that as the doping level of either MCMF or CCMF increases, the SNR improves, while the MAE exhibits an inverse relationship, decreasing with increasing doping levels. A desired performance is characterized here by a high SNR and low MAE, achieved with 0.25 wt% MCMF and 0.25 wt% CCMF (3G250M250CCMF). Specifically, the SNR peaks at 21.98 dB, while the MAE is minimized to 74 με. Specimens 3G250M500CCMF and 3G125M500CCMF also performed well.

### 4.3. Dynamic Cyclic Load Tests

**Figure 8:**
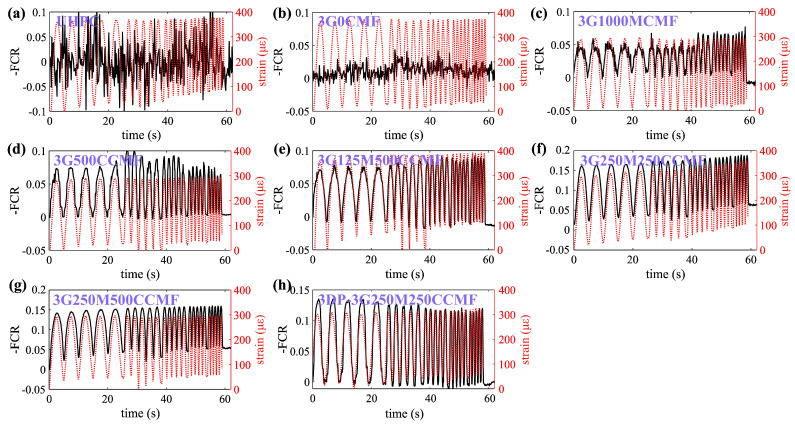
Time histories of electrical and strain measurements under increased loading frequency for specimens (**a**) 0G0CMF; (**b**) 3G0CMF; (**c**) 3G1000MCMF; (**d**) 3D500CCMF; (**e**) 3G125M500CCMF; (**f**) 3G250M250CCMF; (**g**) 3G250M500CCMF; (**h**) 3DP-3G250M250CCMF.

Figure 8a–g presents time series plots for UHPC specimens doped with key mix designs compared against strain input to evaluate the stability of the piezoresistive response under dynamic cyclic loading. Similar to the results obtained from the quasi-static cyclic loading test (Figure 7), the UHPC-only specimen (0G0CMF) also exhibited no discernible piezoresistive response, displaying high levels of noise and no clear correlation between the electrical signal and applied strain. The addition of G in the UHPC specimen (3G0CMF) improved the conductivity of the matrix and reduced noise levels but did not allow the measurement of the dynamic load.

The addition of CMF enhanced the piezoresistive sensitivity of all specimens, with significant improvements in signal fitting and noise reduction compared to the baseline UHPC-only specimen (0G0CMF), as indicated by the consistent cyclic patterns in their FCR signals that closely followed the applied strain levels. Dual-doped specimens exhibited further enhancements in sensing performance compared to single-doped specimens. The 3G125M500CCMF, 3G250M250CCMF, and 3G125M500CCMF specimens displayed the most repeatable and reversible behaviors, with excellent fitting of the applied strain under each frequency. The performance of the 3D-printed specimens also showed a similar trend but with a decreased sensitivity compared to the mold-cast counterparts.

The dynamic strain gauge factor $\lambda_{\mathrm{dyn}}$ at each frequency was computed using the data measured from the dynamic cyclic loading test (Figure 8) by calculating the average power spectral densities of the cyclic loading data and determining the ratio of resistance-to-strain changes. Quantitative results, along with the associated mean (μ) and standard deviation ($\sigma_{\lambda_{\mathrm{dyn}}}$), are presented in Table 6. It can be observed that the dynamic strain gauge factor $\lambda_{\mathrm{dyn}}$ of all specimens remains relatively constant across the tested frequencies of 0.2, 0.4, 0.6, 0.8, and 1 Hz, with acceptable standard deviation ($\sigma_{\lambda_{\mathrm{dyn}}}$) values that remain below or around 20. This suggests that the strain gauge factor is largely independent of frequency over this particular range of frequencies. A comparison of the 3D-printed specimen with its mold-cast counterpart shows a reduction of 7% in the gauge factor, but a net increase in the stability of its gauge factor.

The reduced sensitivity of the 3D-printed specimen, reflected through its lower gauge factor, can be attributed to the interlayer bond and increased porosity introduced by the 3DP process, which can hinder both electrical conductivity and strain transfer across layers. This occurs because the weaker bonding between layers disrupts the conductive network, limiting the continuity of electrical pathways compared to the mold-cast specimen. Additionally, interlayer bonding issues reduce the efficiency of strain transfer, as strain applied to one layer may not be fully transferred to adjacent layers, resulting in a weakened piezoresistive response.

### 4.4. Monotonic Compressive Load Tests

**Figure 9:**
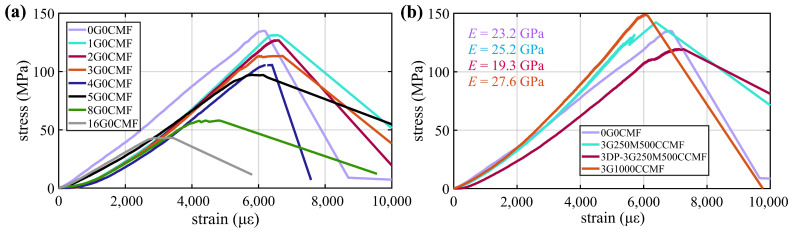
Stress strain curve for (**a**) G dopant; (**b**) selected 3DP versus mold-cast specimens.

Figure 9a compares the 28-day three-specimen averaged stress–strain curves for G-filled specimens with different G inclusion levels. It can be found that the addition of G to the UHPC led to a decrease in compressive strength, defined as the maximum stress attained before failure, here ranging from 2.6% to 68.0% as the G content increased from 1 wt% to 16 wt% compared to the UHPC-only specimen (0G0CMF). Similarly, Young’s modulus (*E*), calculated from the slope of the elastic region indicating the material’s stiffness, exhibited a reduction of 1.7% to 33.8%, while the ultimate strain decreased by 4.6% to 50.1% across the same G content range. These results suggest that the material becomes both less stiff and less ductile with increasing G content. The decrease in these mechanical properties can be attributed to several factors: the lower modulus of elasticity of G, its capacity to absorb water through its pores, interference with UHPC hydration as G particles occupy spaces that would otherwise facilitate the formation of calcium silicate hydrate (C-S-H); weaker bonding at the interface between G particles and the cementitious matrix, and less dense particle packing as the addition of G tends to introduce voids and increase porosity [28].

Figure 9b compares the selected stress–strain curves. It can be observed that the addition of MCMF and CCMF to the UHPC increases mechanical strength. In particular, the 3G250M500CCMF specimen reached a compressive strength of 142.3 MPa and Young’s modulus of 25.2 GPa, which are, respectively, 5.2% and 8.6% higher than those of the UHPC-only specimen (0G0CMF). The compressive strengths and Young’s moduli of the 3DP-3G250M500CCMF specimens were measured as 123.3 MPa and 19.3 GPa, respectively, representing reductions of 13.4% and 23.7% compared to its mold-cast counterparts. This reduction in mechanical properties can be attributed to inherent microstructural differences between the 3DP and casting processes, as the layer-by-layer deposition in 3D printing results in weaker interlayer bonding and increased porosity.

All the specimens, except those with G inclusion exceeding 3 wt%, doped with MCMF, CCMF, and dual MCMF+CCMF exhibited compressive strengths greater than 120 MPa (17,000 psi), meeting the minimum strength requirement for UHPC [58]. The 3G1000CCMF outperformed in mechanical strength with 9.8% and 19.2% improvements in compressive strength and Young’s modulus, respectively, compared to plain UHPC (0G0CMF). These results also align with findings from the literature, indicating that fiber incorporation contributes to the formation of a dense microstructure, resulting in the enhanced compressive strength of UHPC [70]. It should also be noted that the mechanical performance of the 3D-printed self-sensing UHPC specimens was substantially enhanced to that of 3D-printed self-sensing cement paste in our prior study. Specifically, Young’s modulus of the 3D-printed UHPC specimens in this work ranged from 16 to 19 GPa, compared to values of 500 to 800 MPa reported for the 3D-printed cement paste [41].

### 4.5. Sensing Performance

**Figure 10:**
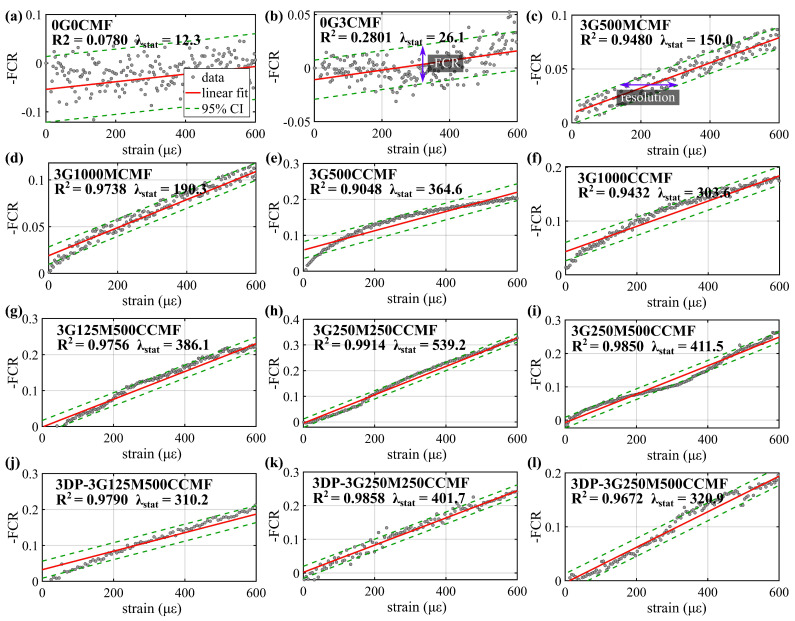
Plots of -FCR measured from the monotonic compressive loading test for specimens under representative mix designs.

Figure 10a–l plots the -FCR versus strain taken from the 0 to 600 με region of the monotonic compressive loading test. This strain range was selected because it is representative of the operational strain in SHM applications, and also because it remains within the elastic region of the material’s behavior, where the piezoresistive response is the most linear and stable, ensuring the repeatability of tests. Plots include a linear fit (red solid line) and its associated 95% confidence interval (CI) bound (dashed green lines). The linearity of the measurements (R^2^) and strain gauge factor ($\lambda_{\mathrm{stat}}$) computed from the slope of the linear fit are identified in each subplot. The presented datasets are taken from the best-performing specimen under each mix design in terms of the linearity of the signal. Table 5 assembles the quantitative three-specimen averaged sensing metrics obtained from Figure 10 under each mix design, where the resolution is defined by the FCR range within the 95% CI and the equivalent strain levels computed using Equation (4), as indicated in Figure 10b,c, respectively, and the accuracy ($\sigma_{\mathrm{res}}$) is computed as the standard derivation of the resolution.

The results show that the UHPC-only specimen (0G0CMF) exhibited a poor piezoresistive response, and the addition of G to the UHPC matrix significantly improved linearity (R^2^), strain sensitivity (λ), and resolution. The single-doped 3G1000MCMF and 3G500CCMF specimens outperformed in their respective category by yielding the highest strain gauge factor (200 and 365, respectively) representing a net improvement over the UHPC-only ($\lambda_{\mathrm{stain}}$ = 13.1) and 3 wt% G-only ($\lambda_{\mathrm{stain}}$ = 29) specimens. These specimens also yielded the best strain resolutions (214 με and 144 με, respectively) in agreement with the ranking of performance with respect to SNR and MSE.

The hybrid mixture 3G250M250CCMF outperformed all mix designs in terms of the strain sensing performance, improving linearity by 9.8%, sensitivity by 380.1%, strain resolution by 68.2%, and accuracy by 68.0% compared to 3G250MCMF, and by 9.58%, 47.9%, 64.0%, and 57.2% compared to 3G250CCMF. Both single-doped mixtures had the same CMF doping level as 3G250M250CCMF but without the hybrid configuration. The 3G250M500CCMF mixture, despite exhibiting lower resistivity than the 3G250M250CCMF specimen due to its higher fiber content, showed a slightly reduced sensing performance compared to 3G250M250CCMF, attributable to fiber agglomeration and/or reduced piezoresistivity. Generally, the ranking of the strain gauge factors $\lambda_{\mathrm{strain}}$ characterized here is consistent with the piezoresistive responses observed during the dynamic cyclic loading test (Figure 8 and Table 6), with discrepancies attributable to the polarization effect found in functionalized materials at lower frequencies [68,71].

The sensing metrics for the 3D-printed specimens 3DP-3G125M500CCMF, 3DP-3G250-M250CCMF, and 3DP-3G250M500CCMF demonstrated similar linearity but exhibited 11.4%, 24.1%, and 19.1% lower static strain gauge factors, along with 10.6%, 31.3%, and 27.4% lower resolution, respectively, compared to their mold-cast counterparts. When compared to the 3D-printed self-sensing cement paste from our prior study, the 3D-printed self-sensing UHPC specimens exhibited slightly lower strain sensitivity but achieved higher resolution and accuracy. Specifically, the best-performing 3D-printed cement paste specimen exhibited a strain gauge factor of 622, a resolution of 167 με, and an accuracy of 19.24 με, whereas the 3DP-3G250M250CCMF yielded a strain gauge factor of 540, a resolution of 68 με, and an accuracy of 4.5 με. This difference is likely attributed to the denser microstructure and higher stiffness of UHPC, which reduced the piezoresistive effect but enhanced the stability of the electrical signal.

Other than the interlayer bonding and increased porosity that can affect the sensing performance for the 3D-printed specimens, as discussed earlier, the shear flow of the extrusion process can provoke an orientation of the CMF along the print direction, thus reducing fiber connectivity in the vertical (build-up) direction where the measurements were taken [72]. This anisotropic alignment of the CMF may further deteriorate the effective transfer of strain and electrical signals in the direction perpendicular to the print layers. This is left to future work.

While out-of-the-scope of this work, it may also be possible to enhance the sensing performance of 3D-printed specimens. A solution would be to strengthen interlayer bonding through the optimization of print parameters, such as extrusion speed, nozzle temperature, and layer height, that may facilitate more uniform layer integration and reduce interface-induced discontinuities that impair signal transmission and strain transfer. Another solution is to modify the material composition by incorporating coupling agents or functional additives that could improve particle connectivity and reinforce interlayer bonding, resulting in a more continuous conductive network. Post-print thermal or mechanical treatments may further promote denser particle packing and interlayer coherence, potentially enhancing strain sensitivity and resolution in 3D-printed specimens.

## 5. Conclusions

This paper presented a study on the fabrication of 3D-printed self-sensing ultra-high-performance concrete (UHPC) composites incorporating graphite (G) powder, milled carbon microfibers (MCMF), and/or chopped carbon microfibers (CCMF) to reach electrical percolation and enhance the piezoresistive properties for strain sensing. UHPC specimens were fabricated using 29 different mix designs, specifically designed to establish the percolation curve and ensure the feasibility of 3D printing. Resistance was measured using a two-probe alternating current with a frequency sweep setup for quality control. Quasi-static cyclic and dynamic cyclic load tests were performed to evaluate the piezoresistive properties in terms of the signal-to-noise ratio, mean absolute error, dynamic gauge factor, and signal stability. Additionally, uniaxial monotonic compressive load tests were conducted to characterize the mechanical properties that examine the materials’ compressive strength and Young’s moduli and to evaluate the sensing performance in terms of signal linearity, static gauge factor, resolution, and accuracy. Based on the analysis of the results, the following conclusions can be drawn:

- The incorporation of G, MCMF, and/or CCMF significantly accelerated electrical percolation, with saturation achieved at a doping level of 3 wt% G combined with either 0.5 wt% MCMF or 0.5 wt% CCMF.
- Piezoresistive sensing performance consistently improved as the inclusion level of MCMF or CCMF increased from 0 wt% to 1 wt%, with the dual MCMF + CCMF combination substantially enhancing the piezoresistive sensitivity of the UHPC composites.
- The addition of MCMF and CCMF slightly increased mechanical properties, with the 3G1000CCMF specimen yielding 9.8% and 19.2% higher compressive strength and Young’s modulus, respectively, compared to the UHPC-only specimen (0G0CMF).
- The 3G250M250CCMF mixture delivered optimal sensing performance under monotonic compressive load tests, resulting in a strain gauge factor ($\lambda_{\mathrm{stat}}$) of 540, a resolution of 68 με, and an accuracy of 4.5 με.
- The 3D-printed UHPC composites achieved a slightly lower mechanical performance and piezoresistive response compared to their mold-cast counterparts, attributed to the inherent microstructural differences induced by the layer-by-layer printing process, yielding a strain gauge factor ($\lambda_{\mathrm{stat}}$) of 410, a resolution of 99 με, and an accuracy of 8.6 με.

The comparison between 3D-printed and mold-cast specimens revealed that the 3D-printed specimens with the top three mix designs demonstrated acceptable piezoresistive performance, with gauge factors marginally lower than those of the mold-cast counterparts. This confirms the viability of using 3D printing to fabricate self-sensing UHPC for SHM applications, although further optimization of the printing parameters is necessary to minimize the porosity and enhance interlayer bonding.

## Figures and Tables

**Table 1:** Typical composition of Ductal^®^ UHPC.

Material	Density (kg/m^3^)	Weight Percentage (%)	Conductivity (S·cm)
Portland cement	712	28.5	3 × 10^−3^
fine sand	1020	40.8	1–2 × 10^−4^
silica fume	231	9.3	2 × 10^−5^
ground quartz	211	8.4	1–3 × 10^−6^
HRWR	30.7	1.2	2–3 × 10^−3^
accelerator	30.0	1.2	4 × 10^−5^
steel fiber	156	6.2	1 × 10^5^
water	1.0	4.4	5 × 10^−2^

**Table 2:** Material properties for the TITGGI G powder and SGL milled and chopped CMF [45].

Material Properties	Units	G Powder (TITGGI 400 Mesh)	MCMF (C M150-4.0/240-UN)	CCMF (C C6-4.0/240-G100)
density	g/cm^3^	1.06	1.80	1.80
mean fiber length	μm	-	150	6000
filament diameter	μm	37	7	7
tensile strength	GPa	-	4.0	4.0
tensile modulus	GPa	-	240	240
elongation at break	%	-	1.7	1.7
single filament resistivity	μΩm	40	15	15
bulk density	g/L	800	250	-
sizing type	-	unsized	unsized	glycerin
sizing mass content	%	-	-	4.0

**Table 3:** Material properties of mixture components. Aspect ratios are taken from literature [25].

Material	Density (g/cm^3^)	Conductivity (S·cm)	Aspect Ratio (-)
UHPC (powder)	2.5	1 × 10^−7^	N/A
AC (powder)	2.8	3–5 × 10^−8^	N/A
G (powder)	1.2	2–3 × 10^3^	≈12
MCMF	1.8	4.3 × 10^4^	≈80
CCMF	1.8	6.5 × 10^4^	≈1300
water	1.0	5 × 10^−2^	N/A
superplasticizer (SP)	1.1	4 × 10^−4^	N/A

**Table 4:** Constitution details of each UHPC specimen.

Mixture Type	G Powder (g)	G/b (%)	MCMF (g)	MCMF/b (%)	CCMF (g)	CCMF/b (%)	Water (g)	w/b (%)	HRWR (g)
0G0CMF	0	0	-	-	-	-	109.5	7.3	15.1
1G0CMF	15	1	-	-	-	-	112.1	7.4	15.1
2G0CMF	30	2	-	-	-	-	114.8	7.5	15.3
3G0CMF	45	3	-	-	-	-	122.1	7.9	15.4
4G0CMF	60	4	-	-	-	-	132.6	8.5	15.6
5G0CMF	75	5	-	-	-	-	144.9	9.2	15.8
8G0CMF	120	8	-	-	-	-	170.1	10.5	16.2
16G0CMF	240	16	-	-	-	-	243.6	14.0	17.4
3G31MCMF	45	3	0.4680	0.03125	-	-	140.6	9.1	15.45
3G63MCMF	45	3	0.9375	0.06250	-	-	140.6	9.1	15.45
3G125MCMF	45	3	1.8750	0.12500	-	-	140.6	9.1	15.45
3G250MCMF	45	3	3.7500	0.25000	-	-	140.6	9.1	15.45
3G500MCMF	45	3	7.5000	0.50000	-	-	140.6	9.1	15.45
3G1000MCMF	45	3	15.0000	1.00000	-	-	140.6	9.1	15.45
3G31CCMF	45	3	-	-	0.4680	0.03125	137.5	8.9	15.45
3G63CCMF	45	3	-	-	0.9375	0.06250	137.5	8.9	15.45
3G125CCMF	45	3	-	-	1.8750	0.12500	137.5	8.9	15.45
3G250CCMF	45	3	-	-	3.7500	0.25000	137.5	8.9	15.45
3G500CCMF	45	3	-	-	7.5000	0.50000	137.5	8.9	15.45
3G1000CCMF	45	3	-	-	15.0000	1.00000	137.5	8.9	15.45
3G63M125CCMF	45	3	0.9375	0.06250	1.8750	0.12500	148.3	9.6	15.45
3G63M250CCMF	45	3	0.9375	0.06250	3.7500	0.25000	148.3	9.6	15.45
3G63M500CCMF	45	3	0.9375	0.06250	7.5000	0.50000	148.3	9.6	15.45
3G125M125CCMF	45	3	1.8750	0.12500	1.8750	0.12500	148.3	9.6	15.45
3G125M250CCMF	45	3	1.8750	0.12500	3.7500	0.25000	148.3	9.6	15.45
3G125M500CCMF	45	3	1.8750	0.12500	7.5000	0.50000	148.3	9.6	15.45
3G250M125CCMF	45	3	3.7500	0.25000	1.8750	0.25000	148.3	9.6	15.45
3G250M250CCMF	45	3	3.7500	0.25000	3.7500	0.50000	148.3	9.6	15.45
3G250M500CCMF	45	3	3.7500	0.25000	7.5000	1.00000	148.3	9.6	15.45
3DP-3G125M500CCMF	45	3	3.7500	0.25000	1.8750	0.25000	148.3	9.6	15.45
3DP-3G250M250CCMF	45	3	3.7500	0.25000	3.7500	0.50000	148.3	9.6	15.45
3DP-3G250M500CCMF	45	3	3.7500	0.25000	7.5000	1.00000	148.3	9.6	15.45

**Table 5:** Averaged strain and stress sensing performance of each mix design.

Mix Design	SNR (dB)	MAE (με)	R^2^ (-)	$\lambda_{\mathrm{stat}}$ (-)	95% CI		$\sigma_{\mathrm{res}}$ (με)
					-FCR	res (με)	
0G0MCMF	0.3	4151	0.08	13	0.133	600+	-
1G0MCMF	0.6	3919	0.11	15	0.108	600+	-
2G0MCMF	1.0	3643	0.17	20	0.071	600+	-
3G0MCMF	1.7	3238	0.28	29	0.036	600+	-
4G0MCMF	2.3	2872	0.26	37	0.039	600+	-
5G0MCMF	3.1	2451	0.31	50	0.041	600+	-
8G0MCMF	4.2	1704	0.51	92	0.052	531	30.0
16G0MCMF	7.4	391	0.91	204	0.033	232	16.1
3G31MCMF	4.0	1372	0.53	49	0.037	600+	-
3G63MCMF	5.0	851	0.70	79	0.041	404	12.5
3G125MCMF	6.4	521	0.81	95	0.034	342	17.2
3G250MCMF	7.3	410	0.90	132	0.029	298	14.1
3G500MCMF	8.7	275	0.95	161	0.019	214	10.9
3G1000MCMF	7.4	236	0.97	200	0.021	178	6.0
3G31CCMF	3.3	1038	0.50	59	0.051	600+	-
3G63CCMF	5.7	706	0.68	80	0.044	432	13.7
3G125CCMF	9.4	423	0.86	184	0.050	390	15.3
3G250CCMF	11.3	222	0.90	260	0.037	304	8.5
3G500CCMF	13.4	131	0.90	365	0.047	144	10.5
3G1000CCMF	9.3	295	0.94	313	0.021	189	6.3
3G63M125CCMF	8.0	340	0.85	173	0.039	333	9.6
3G63M250CCMF	12.1	206	0.89	225	0.037	282	9.2
3G63M500CCMF	13.0	214	0.92	318	0.031	201	5.7
3G125M125CCMF	9.4	228	0.93	262	0.040	241	9.0
3G125M250CCMF	13.9	178	0.95	360	0.029	153	7.9
3G125M500CCMF	17.5	107	0.98	416	0.035	92	5.3
3G250M125CCMF	14.4	154	0.97	348	0.037	189	10.3
3G250M250CCMF	22.0	76	0.99	540	0.032	68	4.5
3G250M500CCMF	19.2	119	0.99	408	0.030	85	5.5
3DP-3G125M500CCMF	15.2	192	0.98	308	0.047	209	11.1
3DP-3G250M250CCMF	20.3	121	0.99	410	0.036	99	12.5
3DP-3G250M500CCMF	18.0	165	0.97	330	0.031	117	8.6

**Table 6:** Dynamic strain gauge factor $\lambda_{\mathrm{dyn}}$ across different loading frequencies, along with the computed mean and standard deviation values.

Mix Design	Frequency (Hz)					μ	$\sigma_{\lambda_{\mathrm{dyn}}}$
	0.2	0.4	0.6	0.8	1.0		
0G0MCMF	10.0	6.7	3.9	3.2	3.7	5.5	2.9
3G0MCMF	16	13	9.5	8.9	8.8	11	3.3
3G1000MCMF	181	173	184	173	174	177	5.0
3G500CCMF	329	342	358	351	304	337	21.3
3G125M500CCMF	407	396	405	377	366	390	18.9
3G250M250CCMF	441	445	452	430	421	438	12.6
3G250M500CCMF	421	432	431	411	401	419	13.3
3DP-3G250M250CCMF	410	416	396	407	405	407	7.3

## Data Availability

The data are available in a publicly accessible repository at the following link: https://www.dropbox.com/home/UHPC%20Data, accessed on 21 November 2024.

## Abbreviations

The following abbreviations are used in this manuscript:

UHPC	Ultra-High Performance Concrete
SHM	Structural Health Monitoring
3DP	3D Printing
HRWR	High-range Water Reducing Admixture
G	Graphite
MCMF	Miller Carbon Microfiber
CCMF	Chopped Carbon Microfiber
CMF	Carbon Microfiber
AC	Alumina Cement
FCR	Fractional Change in Resistivity
SNR	Signal-to-Noise Ratio
MAE	Mean Absolute Error
DAQ	Data Acquisition System
PE	Polyethylene
CI	Confidence Interval
